# Forage Preferences of Southern Black‐Throated Finch ( *Poephila cincta cincta* ) in the Desert Uplands Bioregion of Australia

**DOI:** 10.1002/ece3.74425

**Published:** 2026-10-22

**Authors:** Brad Dreis, John van Osta, Sarah Richardt, Laetitia Smoll, James Guy Castley

**Affiliations:** ^1^ E2M Pty Ltd Milton Queensland Australia; ^2^ School of Environment and Science Griffith University Queensland Australia

**Keywords:** diet analysis, dietary selection preferences, foraging, granivore, nutrient profitability, threatened species, woodland birds

## Abstract

Seasonal patterns of resource availability can influence the dietary behaviours of wildlife and how they utilise the landscape. For threatened species, understanding diet preferences and availability and quality of food is also critical for their conservation. This information is important to allow well‐informed decisions for the protection and management of habitat for these species. We investigate the seasonal dietary preferences of the southern subspecies of the endangered black‐throated finch ( *Poephila cincta cincta* ) in the Desert Uplands bioregion of Queensland over 4 years. We examined their diet composition (*n* = 204 observations) and selection preferences from ground cover surveys within 2 × 1 m plots (*n* = 425). Foraged species availability within the study landscape was quantified from 220 randomly selected vegetation cover plots (1 × 1 m plots × 10) and used to determine Jacobs' selectivity index, a measure of diet selection preference. Spearman's correlation coefficient was tested for the nutritional and physical properties of the seeds of the preferred species against Jacobs' Index to determine factors influencing preferential selection. We identified 46 plant taxa, including 33 grass species, in the diet of black‐throated finch. However, a relatively small subset of these grasses was preferred for consumption due to their higher profitability, reduced handling time and seasonal availability. *Digitaria ciliaris* , *Paspalidium rarum*, and *Panicum effusum* were strongly favoured, especially during the wet season when seeds from these grasses are more available. These preferred species have higher metabolisable energy and crude protein content, and shorter seeds than other grasses that are more abundant in the landscape but are consumed less. *Synthesis*: These findings highlight the importance of maintaining habitats that support the availability of nutritionally valuable grass species to aid conservation efforts for this threatened species. We also demonstrate the importance of identifying seasonal patterns of dietary preferences in response to availability and nutrition parameters to better manage wildlife across landscapes.

## Graphical abstract

Understanding how seasonal food availability shapes diet is essential for conserving threatened species and managing their habitats effectively. This study examined the diet of the endangered southern black‐throated finch in Queensland's Desert Uplands, combining field observations with vegetation and nutritional analyses to assess food preferences. Although finches consumed many plant taxa, they strongly selected a small number of nutritionally rich grass species—particularly *Digitaria ciliaris*, *Paspalidium rarum* and *Panicum effusum*—highlighting the importance of maintaining habitats that support these key resources.

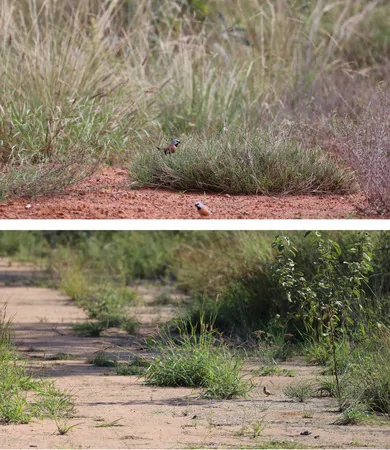

## 1 Introduction

Maintaining habitat quality is essential for the survival, reproduction, and long‐term persistence of wildlife populations (Block and Brennan 1993; Johnson 2007). The nutritional quality and availability of food sources are critical components of habitat quality, as they play pivotal roles in offspring development and adult fitness (Martin 1987; Briga et al. 2017; Mishra and Kumar 2019). Poor nutrition can result in reduced body size, lower survival rates, diminished fecundity, and compromised reproductive success (Metcalfe and Monaghan 2001; Maute 2011; Smith et al. 2019). The nutritional quality of food sources also varies across spatial and temporal scales (e.g., Brown and Nocera 2017; Stone et al. 2019) and is often significantly altered by environmental disturbances such as fire (Bennett et al. 2003), with subsequent consequences for habitat selection by species (Weier et al. 2017). Moreover, food sources vary in abundance, and animals may exhibit selective feeding behaviour by consuming certain co‐occurring foods at disproportionately higher rates than others (Jacobs 1974). In addition to the quantity of food sources, the nutritional composition of seeds may influence habitat selection for granivorous birds (Ríos et al. 2012; Langlois and McWilliams 2022). Consequently, a comprehensive understanding of the foraging ecology of species, particularly those that are threatened, is critical for their conservation. This requires collecting observational data on the foraging behaviours of wildlife, as well as information on the broader availability of their preferred food sources (Sutherland 2004).

Optimal foraging theory predicts that animals seek to maximise their energy gain relative to the effort spent (MacArthur and Pianka 1966). When food is unevenly distributed in patches (i.e., resource locations within habitat), animals must consider travel time, food type and food density in each patch, as well as caloric and nutritional intake and predation risk (Cowie 1977; Benkman 1991; Alonso et al. 1995; Sauter et al. 2006; King and Marshall 2022). The profitability of food therefore strongly influences the behaviour of animals who maximise their energy intake by selecting food items that provide the most calories and nutrients relative to handling time, which is influenced by time to process (Wiens 1989; Díaz 1990, 1996; Marone et al. 2022) and time spent foraging (Pyke 1984; King and Marshall 2022). Strategies for efficient foraging therefore must consider the trade‐off between the size, handling time and availability of any food resource (Werner and Hall 1974; Grundel 1992; Sauter et al. 2006). More profitable food sources are preferred as they allow any remaining time to be used for other activities including nest construction, avoiding predators or defending territory.

Within the framework of optimal foraging theory, diet preferences within granivorous birds are a function of the interaction between the profitability of the food source (i.e., the calories or energy gain relative to handling time), its availability throughout a foraging landscape and its handling efficiency, based on the physical properties of the seed, including its size (Schluter 1982; Kohlmann and Risenhoover 1996; Molokwu et al. 2011; Geiger et al. 2014; Titulaer et al. 2018). The quality and availability of food resources strongly influences the foraging behaviours of granivorous birds (Schluter 1982). When food is plentiful, foraging time typically decreases. However, when food is scarce, as observed for long‐tailed finches ( *Poephila acuticauda* ) and masked finches ( *Poephila personata* ), feeding rates have been observed that would necessitate approximately six hours of foraging per day (Garnett and Crowley 1994). Studies of Darwin's finches (*Geospiza* spp.) found that birds selected seeds with low energy returns where they occurred in high density, therefore reducing energetic costs associated with searching for more profitable food sources (Schluter 1982).

A time‐minimisation strategy relevant to granivorous birds is to select seeds that align with their morphological adaptations so as to minimise handling and processing times (Willson and Harmeson 1973; Keating et al. 1992; Nagy Koves Hrabar and Perrin 2002; Carrillo et al. 2007; Titulaer et al. 2018). Birds with smaller bills may process small seeds faster than larger‐billed birds due to their increased dexterity in positioning the seeds for dehusking or cracking (Díaz 1990), while birds with larger bills can handle larger seeds more efficiently as well as being able to generate more force to crack seeds open (Van der Meij et al. 2004). Consequently, certain bill sizes and morphologies optimise the processing speed for specific seeds (Schluter 1982; Grant 1986; Díaz 1990, 1996), and selecting seeds that are optimal for an animal's morphology can help minimise the time spent foraging.

While seed selection based on morphological characteristics and handling time has been shown to be an important strategy for granivorous birds (Molokwu et al. 2011), research shows that the nutrient content of seeds also plays a role in diet choice (Díaz 1996; Schaefer et al. 2003; Valera et al. 2005; Carrillo et al. 2007; Molokwu et al. 2011; Ríos et al. 2012; Weier et al. 2017). Grass seeds provide the energy required by granivorous birds through various nutritional components including carbohydrates, protein and other compounds (Gluck 1985; Díaz 1996; Carrillo et al. 2007; Brzęk et al. 2010; Ríos et al. 2012). Protein also contributes to growth and development, reproductive success, and stress and immune responses in birds (Pryke et al. 2012).

Granivorous birds inhabiting savannas in northern Australian encounter seasonal fluctuations in the availability of food sources (Woinarski and Tidemann 1991; Dostine and Franklin 2002). Native annual grasses produce large quantities of dormant seeds during the wet season that provide abundant food resources for granivorous birds (Mott 1978; Andrew and Mott 1983; Crowley and Garnett 1999; Franklin et al. 2005). The availability of grass seeds steadily declines over the dry season due to consumption by granivores and fire (Woinarski and Tidemann 1991) and may be limiting in some areas, resulting in increased stress levels and lower body condition of granivorous birds (Maute 2011). The majority of viable grass seeds germinate soon after the onset of rainfall during warmer temperatures (Andrew and Mott 1983), leading to a rapid decline in the availability of grass seeds in the early wet season (Woinarski and Tidemann 1991; Crowley and Garnett 1999; Dostine and Franklin 2002). This may require birds to consume other food sources and spend prolonged periods of time foraging (Garnett and Crowley 1994; Dostine and Franklin 2002). It is apparent that dietary studies need to account for these seasonal fluctuations to fully understand avian granivore requirements (Franklin et al. 2005), particularly those species that may be threatened.

In Australia, the black‐throated finch (southern subspecies), a threatened woodland granivore, has experienced a large reduction in range and abundance over the last 30 years (Black‐throated Finch Recovery Team 2007; Department of the Environment, Water, Heritage and the Arts 2009). The decline of the black‐throated finch is attributed to a combination of habitat clearing and fragmentation as well as changes in land use, particularly pastoralism and agriculture that have rendered other extant black‐throated finch habitat unsuitable due to the introduction of exotic pasture grasses and altered fire regimes (Black‐throated Finch Recovery Team 2007; Department of the Environment, Water, Heritage and the Arts 2009; Vanderduys et al. 2016; Mula Laguna et al. 2019; Reside et al. 2019). Given the significant alteration of black‐throated finch foraging habitat, it is a suitable focal species to enhance our understanding of their foraging behaviour and dietary preferences, including seasonal variations, to effectively inform conservation and management strategies.

This study aimed to identify the broadscale dietary preferences of the black‐throated finch in the Desert Uplands Bioregion of Queensland, Australia, and determine whether they preferentially select food resources within suitable habitats based on seasonal availability, profitability and/or seed morphology. To achieve this, we (i) investigated the diet of black‐throated finches through field observations of foraging activity and habitat assessments at foraging locations, (ii) compared these foraging findings across seasons and against the composition of vegetation in the broader landscape of predicted habitat to determine whether they display any preferences for specific grass species; and (iii) analysed the nutritional content and morphological properties of grass seeds to determine whether black‐throated finches select their preferred food due to profitability or seed morphology. By understanding the foraging behaviour and dietary requirements of the black‐throated finch, the results of this work will contribute to the development and implementation of management strategies to aid the persistence of threatened avian granivore communities more broadly.

## 2 Methods

### 2.1 Study Area and Black‐Throated Finch

The study was undertaken over approximately 70,000 ha centred on 21°59′ S, 146°21′ E and located approximately 150 km north‐west of Clermont and 190 km south of Charters Towers (Figure 1), within the Alice Tablelands subregion of the Desert Uplands Bioregion in Central Queensland, Australia. The Desert Uplands cover an area of approximately 70,000 km^2^ and are characterised by sandstone ranges and sand plains with low‐fertility soils and eucalypt and acacia woodlands with a spinifex understorey. The region has a semi‐arid climate with variable annual rainfall of approximately 460 mm, and the predominant land use in the region is cattle grazing, with some sheep grazing in the west (Sattler and Williams 1999; Bastin et al. 2009). The Desert Uplands Bioregion coincides with the southern extent of the current known range of the black‐throated finch as well as the Galilee Basin where more than 50% of potential black‐throated finch habitat occurs under mining leases (Vanderduys et al. 2016; Reside et al. 2019).

The study area is 63,560 ha and is characterised by cattle grazing and native vegetation, of which approximately 33,400 ha is managed as an environmental offset under the Commonwealth *Environment Protection and Biodiversity Protection Act 1999*, and approximately 33,660 ha consists of a portion of the Carmichael Coal Mine mining lease (e2m 2025). Active mining locations, which are currently approximately 3500 ha, have been excluded from the study area. Approximately 79% of the study area consists of remnant vegetation, comprising 24 vegetation communities described as Regional Ecosystems (REs) (Neldner et al. 2022). The vegetation is dominated by eucalypt woodlands, particularly Regional Ecosystems 10.5.5a ( *Eucalyptus melanophloia* woodland on plains) and 10.5.1 (*Eucalyptus similis* or *Corymbia brachycarpa*, or both woodland on sand plains) that account for 73.5% of the remnant vegetation within the study area (e2m 2025). Other habitats include acacia woodlands and shrublands, with approximately 10,207 ha of the study area cleared for grazing pastures, roads, and mining infrastructure (e2m 2025). Darkies Range borders the western extent of the study area, with one major watercourse, the Carmichael River, and several other smaller creeks including North Creek and Tomahawk Creek.

There is limited knowledge of the ecology, including the diet, of the black‐throated finch from the Desert Uplands region, with previous studies focusing attention on the Townsville Plains (Mitchell 1996; Rechetelo et al. 2016; Mula Laguna et al. 2019; Williams et al. 2020) and Atherton Tablelands (Zann 1976). These studies have revealed that the black‐throated finch consumes seeds from a variety of grasses and legumes, with birds selectively targeting different species across seasons (Zann 1976; Mitchell 1996; Isles 2007). However, these studies are unlikely to be meaningful for understanding the foraging ecology of the black‐throated finch in the Desert Uplands due to differences in habitats, vegetation composition, climate and the availability of water between the two regions (Sattler and Williams 1999; Bastin et al. 2009).

While black‐throated finches have been observed taking seed directly from grass inflorescences (Zann 1976; Mitchell 1996), they spend much of their time foraging directly from the soil surface where they eat fallen and half‐ripe grass seeds (Zann 1976; Mitchell 1996; Rechetelo 2015), making it difficult to determine what species of grass seeds they are consuming. In the Desert Uplands region, which has distinct wet (December to May) and dry seasons (June to November) (Sattler and Williams 1999), the availability of native grass seeds fluctuates significantly between seasons. Black‐throated finches are therefore likely to adjust their diet throughout the year in response to these seasonal changes in seed availability, similar to other granivorous birds in northern Australia (Garnett and Crowley 1994; Dostine and Franklin 2002).

**FIGURE 1:**
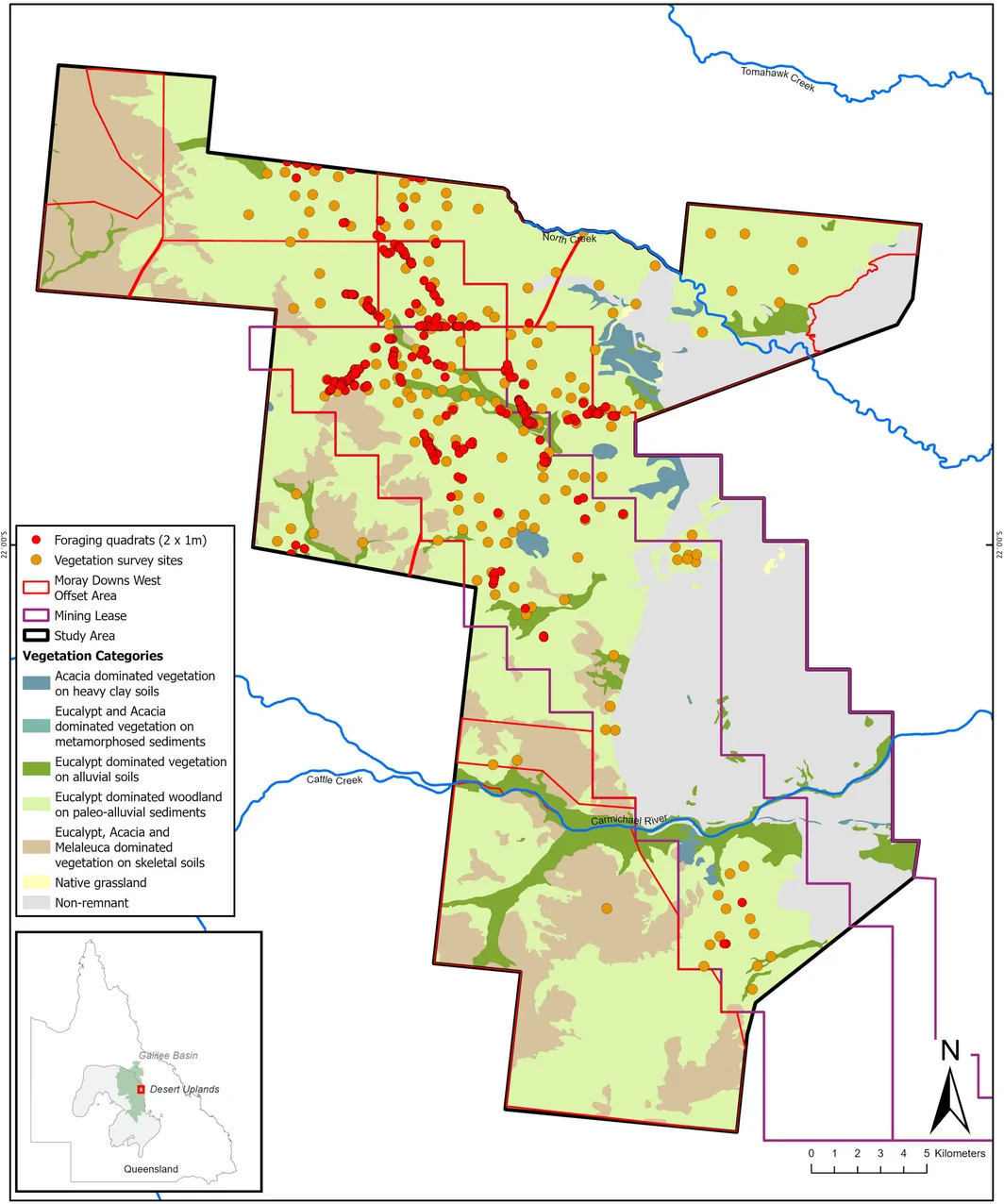
Study area for black‐throated finch research undertaken within the Desert Uplands bioregion of Queensland. The map shows the locations of 220 vegetation survey sites and 425 foraging quadrats where black‐throated finches were observed foraging, vegetation communities, major watercourses, mining lease and offset area.

### 2.2 Survey Design

A total of eight seasonal surveys were conducted over a four‐year period between March 2020 and November 2023. Surveys were undertaken biennially in each year, comprising one survey during the wet season (December to May) and one survey during the dry season (June to November) (Woinarski et al. 2020), to evaluate seasonal variation in diet and resource availability. Each survey period was undertaken over a four to six‐week period. Surveys involved the collection of direct observational data on black‐throated finch foraging behaviour, vegetation ground cover and seed collection for the analysis of seed morphology and quality. The methods for data collection and analysis for each data type are described below.

### 2.3 Foraging Observations

Where foraging black‐throated finches were encountered, we observed their behaviour and, where birds were foraging directly from the plant, we recorded the species which they were consuming. We recorded 204 foraging observations, the majority of which were recorded in the wet season (*n* = 182), when birds often forage directly from the grass inflorescence, facilitating identification of the forage species. During the dry season, identification of the food source was more difficult during field observations as grasses had typically dropped all seed and birds largely forage from seeds on the ground. For each observation of black‐throated finch foraging we recorded the grass species upon which the black‐throated finches were observed to be foraging.

### 2.4 Vegetation Sampling

#### 2.4.1 Bird Foraging Sites

At locations where black‐throated finches were observed foraging, vegetation plots were used to determine the composition and cover of the ground layer at each foraging site. At these targeted sampling sites, we placed a 2 × 1 m quadrat centred on the location where birds were feeding, for a total of 396 groundcover plots. This includes 192 plots assessed where birds were observed foraging but no direct observation of the specific foraging species was recorded (e.g., birds flew off before the target species could be identified or birds were foraging directly from the ground). A single larger plot was used for the bird foraging plots due to the behaviour of the birds which would move during foraging. The 2 × 1 m plot size provided a better representation of the ground cover composition and structure at the location where each bird was observed foraging.

#### 2.4.2 Landscape Forage Availability

To assess the availability of foraged species across the study landscape, we surveyed 220 sites that were randomly stratified within the study area based on the modelled relative habitat suitability for the black‐throated finch. Sites were selected using the Create Spatial Sampling Locations tool in ArcGIS, within areas of eucalypt woodland habitat based on a MaxEnt model developed for black‐throated finch relative habitat suitability within the study area. Areas of unsuitable black‐throated finch habitat, such as cleared pasture and areas with a low suitability (< 0.25) MaxEnt score, were excluded from the sampling design. At each of these sites, ten 1 × 1 m quadrats along a 100 m transect were analysed to characterise the composition and availability of groundcover across the study area.

For all vegetation quadrats outlined above we recorded: (i) grass species richness, (ii) estimated projected foliage cover of each ground cover species, (iii) estimated projected foliage cover of each shrub species, (iv) organic litter cover, (v) bare ground cover, (vi) rock cover, (vii) coarse woody debris cover; and (viii) seed abundance and species of seeding plants.

### 2.5 Seed Nutritional Content and Morphology

Seeds were collected directly from the individual grass species black‐throated finches were observed consuming, and other grasses that are widespread and abundant in the study area in proximity to foraging locations. Seeds were stored in paper bags for up to a week before being dried in an electric fan‐forced drier at 60°C for 12 h and dehusked (Crowley and Garnett 2001; Stock et al. 2013). Following dehusking, samples where sufficient seed mass could be collected (at least 10 g dried weight), were collected from 36 grass species between 2021 and 2022 and placed in sealed vials. Samples were stored for up to two weeks before being sent to the NSW Department of Primary Industries for nutritional content analysis at the completion of the field surveys. Following the methodology outlined by Weier et al. (2017), the following data were recorded, for each sample: (i) total dry weight, (ii) crude protein, (iii) crude fat, (iv) fibre, (v) water‐soluble carbohydrates, (vi) ash content, (vii) metabolisable energy, (viii) carbohydrate content; and (ix) amino acid suite (including threonine and tyrosine, which are essential amino acids that must be obtained directly from seeds; Weier et al. 2017).

Four seeds from each of 28 grass species were also measured for length (mm) and width (mm). An additional 12 species' mean length were collated from the AusGrass2 database (Simon and Alfonso 2011). A subsample of seeds from the 28 grass species was weighed using digital scales to determine the mean weight (g) of seeds to the nearest 0.00001 g. This subset of species was those that had sufficient seed remaining after nutrient content analysis due to the methodology's destructive nature.

### 2.6 Ethics Statement

All surveys were conducted under the animal ethics permit CA 2020/07/1392, issued by the Queensland Department of Agriculture and Fisheries; research permit WA0025814, issued by the Queensland Department of Environment, Science and Innovation; and Australian Bird and Bat Banding Scheme (ABBBS) authority 2832–01, issued by the Australian Government Department of Climate Change, Energy, the Environment and Water.

### 2.7 Statistical Analyses

Foraging plots and randomly sampled vegetation survey plots were compared in terms of the relative abundance of grass species found to be important in the black‐throated finch diet or prevalent in foraging areas using Mann–Whitney U tests. Data distributions were assessed using Shapiro–Wilk tests within species and survey types. Due to frequent zero variance, non‐normal distributions, bounded percentage data, and variable sample sizes across species, non‐parametric Mann–Whitney U tests were applied consistently to all species.

#### 2.7.1 Diet Selection Preference

To determine whether black‐throated finches in the Desert Uplands preferentially select particular grass species to forage we assessed the availability of food sources across the study area and the contribution of those species to the diet (Jacobs 1974; Dierschke 2002; Magome et al. 2008). An availability index for each food source was calculated by dividing the total cover for each taxon by the total cover for all taxa recorded at 220 vegetation survey sites assessed across the study area. We then calculated a selectivity index for each food source as the proportion of total foraging observations attributable to each taxon. Diet selection preference was then calculated using Jacobs' selectivity index (Jacobs 1974):

$$D=\left(r-p\right)/\left(r+p-2r\times p\right)$$

where *D* = Jacobs' selectivity index, *r* = selection index and *p* = availability index.

Jacobs' selectivity index values range from −1 to 1 where 1 is strong preference and −1 indicates avoidance.

#### 2.7.2 Analysis of Factors Influencing Diet Selection

To determine the factors that may be influencing the preferential selection of grass taxa by black‐throated finches, we calculated the Spearman's correlation coefficient for the nutritional and physical properties of the seeds of 19 grass species against the Jacobs' Index. These were the only species for which the full set of nutritional and physical properties was available, and the Jacobs' Index could be calculated.

## 3 Results

### 3.1 Characteristics of Black‐Throated Finch Foraging Locations

A total of 337 grass species were identified in the 425 foraging plots and 220 systematically collected vegetation plots combined. Grass species recorded in foraging and vegetation plots numbered 115 and 329, respectively, with 107 species that occurred in both.

Foraging plots differed significantly from systematic vegetation survey plots in terms of the average ground cover of several types of grasses. More specifically, foraging plots had significantly higher mean cover of *Digitaria brownii* (cotton panic grass), *D. ciliaris* (summer grass), *Panicum effusum* (hairy panic grass), *Paspalidium rarum* (rare grass), *Enneapogon virens* and *Urochloa subquadripara* (para grass) (Table 1). Mean bare ground cover was also significantly higher in foraging plots (54.4% ± 20.8%) than in vegetation plots (22.7% ± 12.7%; U = 2,218,653; *p* < 0.0001). In contrast, average cover of *Cenchrus ciliaris* (buffel grass), *Heteropogon contortus* (black spear grass), *Sehima nervosum* (white grass) and *Themeda triandra* (kangaroo grass) was significantly lower in foraging plots than in systematically collected vegetation plots (Table 1, Figure 2).

**TABLE 1:** Summary statistics (number of sites, mean percentage cover and standard deviation) and comparison (Mann–Whitney U) of the cover of important grass species in foraging and systematically collected vegetation survey plots.

Species	Foraging			Systematic			*U* statistic	*p*
	*n*	Mean	SD	*n*	Mean	SD		
*Aristida pruinosa*	18	5.5	5.9	61	5.4	4.4	1380	0.94
*Aristida* sp.	64	6.2	6.0	100	8.7	8.5	11,110	0.15
Cenchrus ciliaris*	78	8.4	9.9	160	12.4	11.1	27,242	< 0.001
*Chrysopogon fallax*	84	5.3	5.1	164	4.8	4.2	38,835	0.07
*Digitaria brownii*	57	6.5	5.8	152	5.1	4.9	18,914	0.01
Digitaria ciliaris*	162	11.0	11.7	23	5.0	6.2	4926	< 0.0001
*Enneapogon virens*	75	8.1	6.5	141	4.8	4.0	33,067	< 0.0001
*Eriachne mucronata*	23	8.4	5.4	117	11.7	10.7	6982	0.4
*Heteropogon contortus*	121	9.2	10.9	178	12.0	10.5	69,176	< 0.0001
*Panicum effusum*	89	5.5	5.6	132	4.8	6.2	26,712	< 0.001
*Paspalidium rarum*	77	7.8	7.4	16	1.2	0.9	1778	< 0.0001
*Schizachyrium fragile*	21	5.3	3.9	68	9.3	10.6	2553	0.87
*Sehima nervosum*	22	7.0	8.5	111	11.1	9.7	3968	0.03
*Themeda triandra*	33	6.2	7.7	168	11.7	10.4	12,031	< 0.0001
Urochloa subquadripara*	20	5.4	3.8	4	1.1	1.7	72	0.01

*Note:* Non‐native species depicted with an asterisk.

**FIGURE 2:**
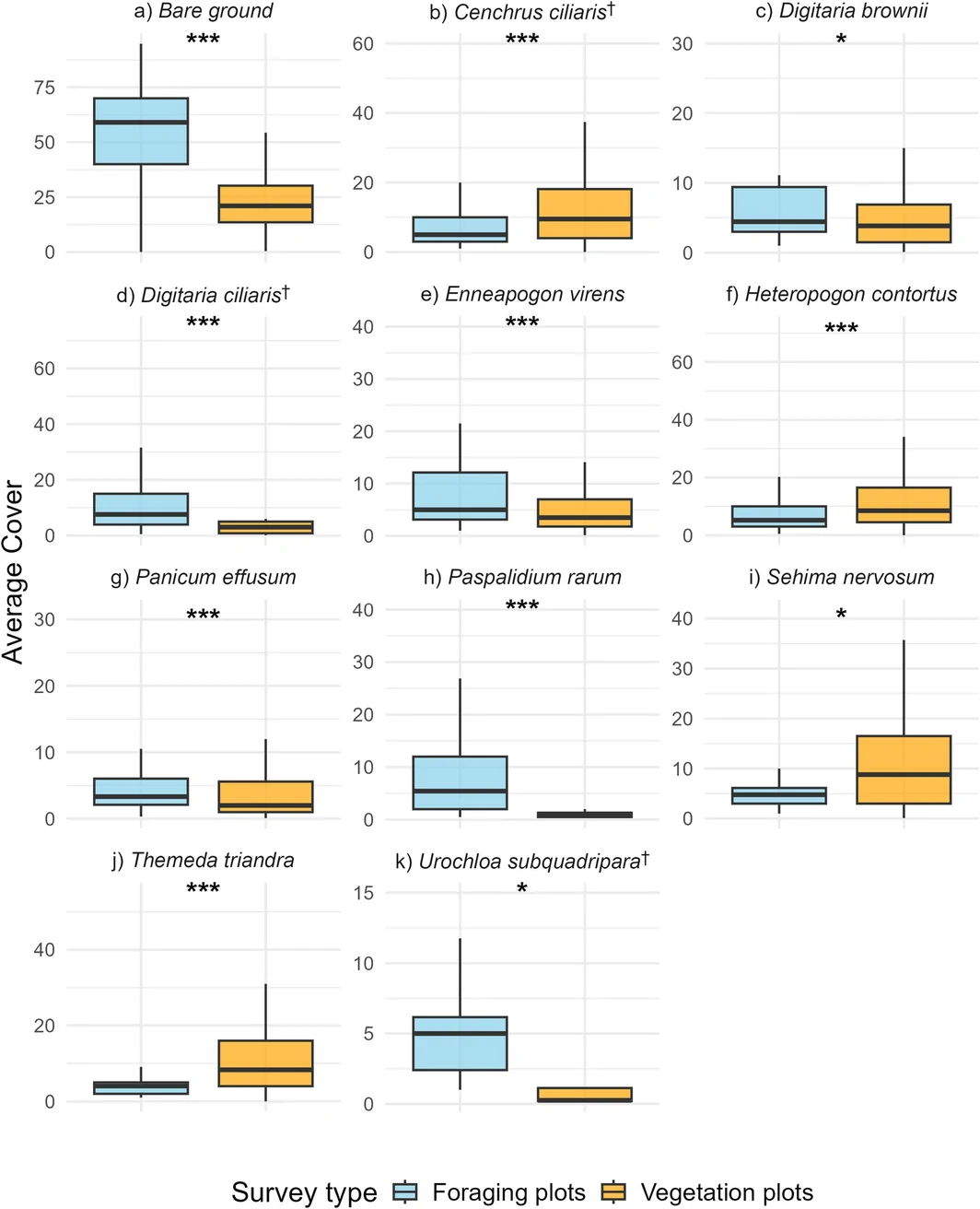
Mean cover (%) of grass species and bare ground recorded at black‐throated finch foraging sites (blue) and systematic vegetation plots (orange) across the study area: (a) bare ground; (b) *Cenchrus ciliaris* ; (c) *Digitaria brownii* ; (d) *Digitaria ciliaris* ; (e) *Enneapogon virens*; (f) *Heteropogon contortus* ; (g) *Panicum effusum*; (h) *Paspalidium rarum*; (i) *Sehima nervosum* ; (j) *Themeda triandra* ; and (k) *Urochloa subquadripara* . Statistical significance is indicated as follows: *p* < 0.05 *, *p* < 0.01 **, *p* < 0.001 ***. Non‐native species depicted with †.

### 3.2 Diet Composition

#### 3.2.1 Foraging Observations

During field observations black‐throated finches were observed foraging directly from grasses, mainly during the wet season, often jumping up from the ground to pull the seed head to the ground and pecking the seed head while being held down by their foot. Out of the 425 observations of birds foraging, 204 observations were able to identify the specific species being foraged. A total of 33 grass species were identified in these observations, the most common forage species being *Digitaria ciliaris* (33.3% of observations), *Paspalidium rarum* (12.9%), *Panicum effusum* (5.3%), *Digitaria brownii* (4.5%), *Cenchrus ciliaris* (4.2%), *Urochloa mosambicensis* (3.8%) and *Urochloa subquadripara* (3.8%) (Table 2). Of these, only *Digitaria ciliaris* and *Digitaria brownii* were observed being foraged on during the dry season, when birds would typically feed on fallen seeds from the ground and grasses were not usually holding seeds. Overall, only about one third of the species that the birds fed on during the wet season (*n* = 31) were fed on in the dry season (*n* = 11) based on these observations.

**TABLE 2:** The number of occasions that southern black‐throated finches were observed foraging on 33 grass species during 204 foraging observations in wet and dry seasons within the study area between 2021 and 2023.

Species	Number of times each grass species was observed being foraged			Overall percentage
	Wet season	Dry season	Total	
Digitaria ciliaris*	78	10	88	33.3
*Paspalidium rarum*	34	0	34	12.9
*Panicum effusum*	14	0	14	5.3
*Digitaria brownii*	9	3	12	4.5
Cenchrus ciliaris*	11	0	11	4.2
Urochloa mosambicensis*	10	0	10	3.8
Urochloa subquadripara*	10	0	10	3.8
*Mnesithea formosa*	8	0	8	3.0
*Dactyloctenium radulans*	7	0	7	2.7
*Enneapogon virens*	5	2	7	2.7
*Tragus australianus*	4	3	7	2.7
*Alloteropsis semialata*	5	0	5	1.9
*Chrysopogon fallax*	5	0	5	1.9
*Schizachyrium fragile*	5	0	5	1.9
*Sehima nervosum*	4	1	5	1.9
*Digitaria ammophila*	3	1	4	1.5
*Aristida pruinosa*	1	2	3	1.1
*Aristida* sp.	2	1	3	1.1
Bothriochloa pertusa*	2	1	3	1.1
*Enneapogon* sp.	3	0	3	1.1
Echinochloa colona*	2	0	2	0.8
*Eragrostis cumingii*	2	0	2	0.8
*Eriachne mucronata*	0	2	2	0.8
*Paspalidium* sp.	2	0	2	0.8
*Urochloa* sp.	2	0	2	0.8
*Whiteochloa airoides*	2	0	2	0.8
*Aristida calycina*	0	1	1	0.4
*Aristida jerichoensis*	1	0	1	0.4
Melinis repens*	1	0	1	0.4
*Setaria surgens*	1	0	1	0.4
*Themeda avenacea*	1	0	1	0.4
*Themeda triandra*	1	0	1	0.4
*Urochloa holosericea*	1	0	1	0.4
Total No. of observations	236	27	263	100
Total species richness	31	11	33	

*Note:* For some observations, birds were observed foraging on multiple species. Non‐native species depicted with an asterisk.

One observation of opportunistic nectarivory by a black‐throated finch was recorded during the 2022 wet season. Six black‐throated finches were photographed and filmed actively obtaining nectar from the open flowers of a *Grevillea pteridifolia*. The foraging lasted for approximately 5 min, after which the flowers of the *Grevillea pteridifolia* were inspected. No insects were observed on the flowers and an abundance of nectar was present, suggesting the black‐throated finches were targeting nectar available in the open flowers.

### 3.3 Diet Selection Preferences

The Jacobs' Index was calculated based on foraging observations (wet season only) to determine whether black‐throated finches preferentially selected specific grass species and taxa.

#### 3.3.1 Selection Preferences Based on Foraging Observations

The Jacobs' Index was calculated for 28 grass species on which black‐throated finches were observed foraging during 231 occasions during the wet season (Jacobs' Index could not be computed for *Echinochloa colona, Paspalidium* sp., *Urochloa holosericea* as these were not recorded in systematically collected vegetation survey sites, preventing the calculation of an availability measure) (Figure 3). *Paspalidium* sp. is likely to have been *Paspalidium rarum* for which Jacobs' Index was calculated, and the other three species were only observed being consumed once or twice. The majority (*n* = 24) of these species had a positive Jacobs' Index, with 14 showing a strong preference (index > 0.8). Only four grass species were avoided, where *Themeda triandra* (−0.85) was actively avoided (Figure 3). Grass species with numerous foraging observations and high Jacobs' Index were *Digitaria ciliaris* (Jacobs' Index = 0.99), *Paspalidium rarum* (0.99), *Urochloa subquadripara* (0.99) and *Panicum effusum* (0.68) (Figure 3). Other species with high Jacobs' Index, such as *Alloteropsis semialata* (0.96), *Eragrostis cumingii* (0.93) and *Melinis repens* (0.95) had very few foraging observations and the high Jacobs' Index is due to a very low availability index.

**FIGURE 3:**
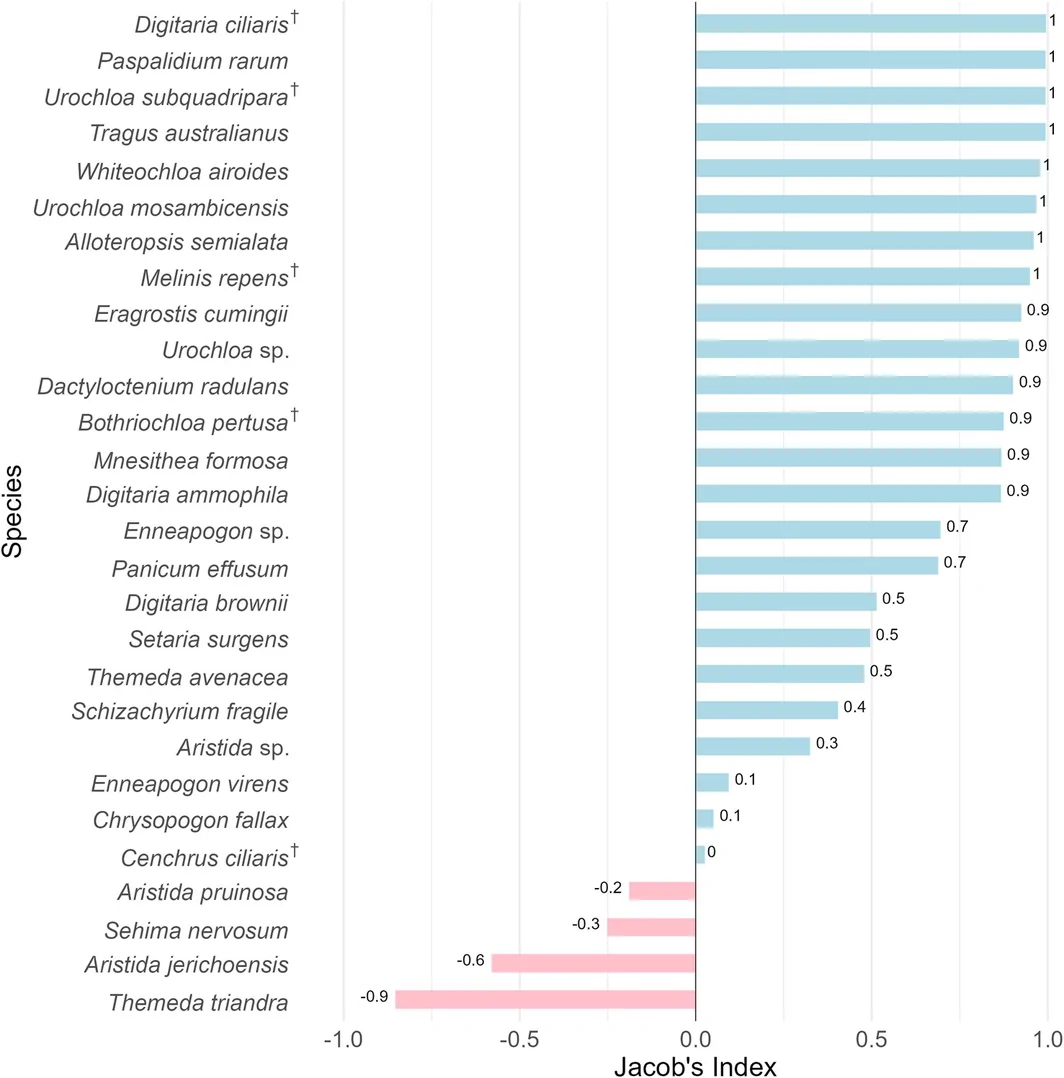
Jacobs' Index calculated for 28 grass species that black‐throated finches were observed foraging on during the wet season between 2021 and 2023. Non‐native species depicted with †.

### 3.4 Factors Driving the Selection of Black‐Throated Finch

To determine the factors that influence why black‐throated finches prefer specific grasses, we analysed the nutritional content and morphology of grass seeds.

#### 3.4.1 Seed Nutritional Content

The results of the seed nutritional analyses identified a relationship between the preferred forage species of black‐throated finches and the concentration of key nutritional factors provided by their seeds. Specifically, metabolisable energy (Spearman's rho = 0.62, *p* = 0.005; Figure 5) and crude protein (rho = 0.50; *p* = 0.03; Figure 6) were positively correlated to the Jacobs' Index of grass species.

Grass seeds containing the highest metabolisable energy content include several preferred diet species: *Digitaria ciliaris* , *Urochloa mosambicensis* , *Panicum effusum* and *Paspalidium rarum* (Figure 4). Most notably, *Digitaria ciliaris* had by far the highest metabolisable energy (11.5 MJ/kg of dry matter). Grasses with higher Jacobs' Index typically contained metabolisable energy levels higher than 7 MJ/kg dry matter, while species with a smaller Jacobs' Index were mostly lower (Figure 4).

Similarly, the grasses with a higher Jacobs' Index also contain higher levels of crude protein. *Digitaria ciliaris* (13.8% of dry matter) also has the highest crude protein content of the grasses that were analysed. Other grass species with high concentrations of crude protein and high Jacobs' Index include *Digitaria ammophila*, *Urochloa mosambicensis* and *Paspalidium rarum* (Figure 5).

**FIGURE 4:**
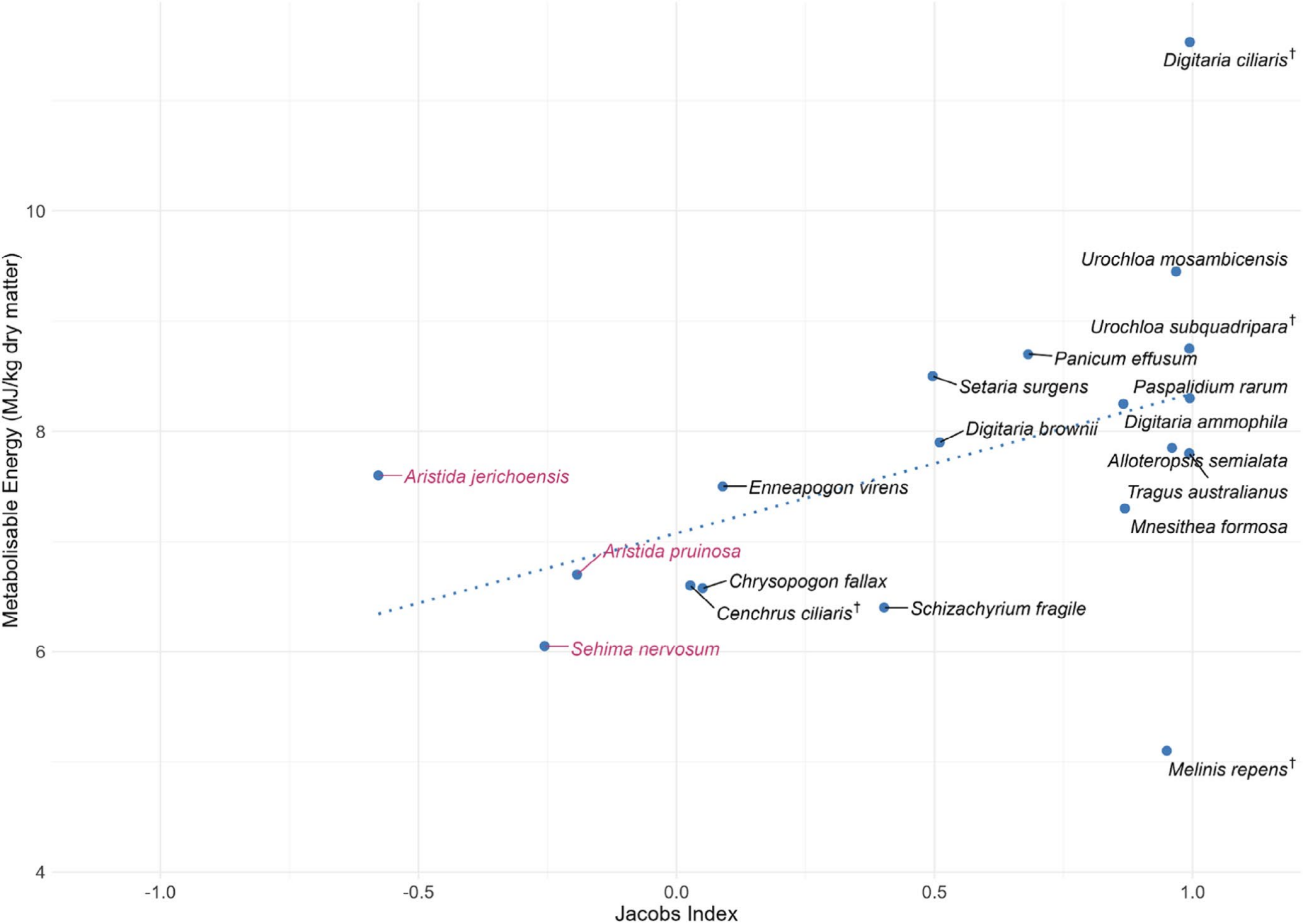
Relationship between Jacobs' Index and metabolisable energy content of the seeds of 19 grass species that occur in the study area. Non‐native species depicted with †. Species with a negative Jacobs' Index are in red font.

**FIGURE 5:**
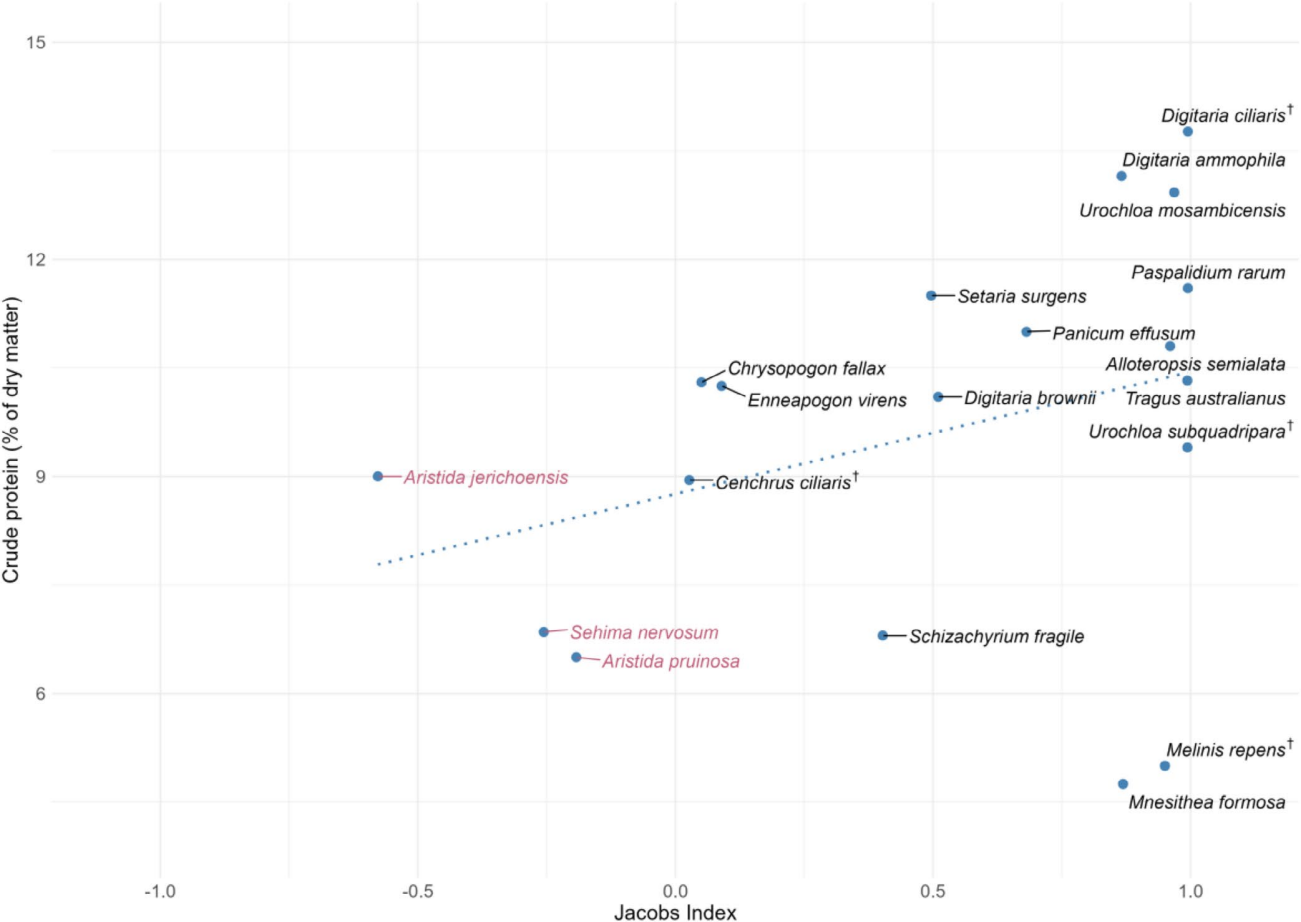
Relationship between Jacobs' Index and crude protein content of the seeds of 19 grass species that occur in the study area. Non‐native species depicted with †. Species with a negative Jacobs' Index are in red font.

#### 3.4.2 Seed Morphology

A negative correlation was identified between preferred forage species of black‐throated finches and the length of seeds (rho = −0.56; *p* = 0.009; Figure 6), indicating that smaller seeds are preferred. However, no relationship was found with seed width (rho = −0.18; *p* = 0.436).

**FIGURE 6:**
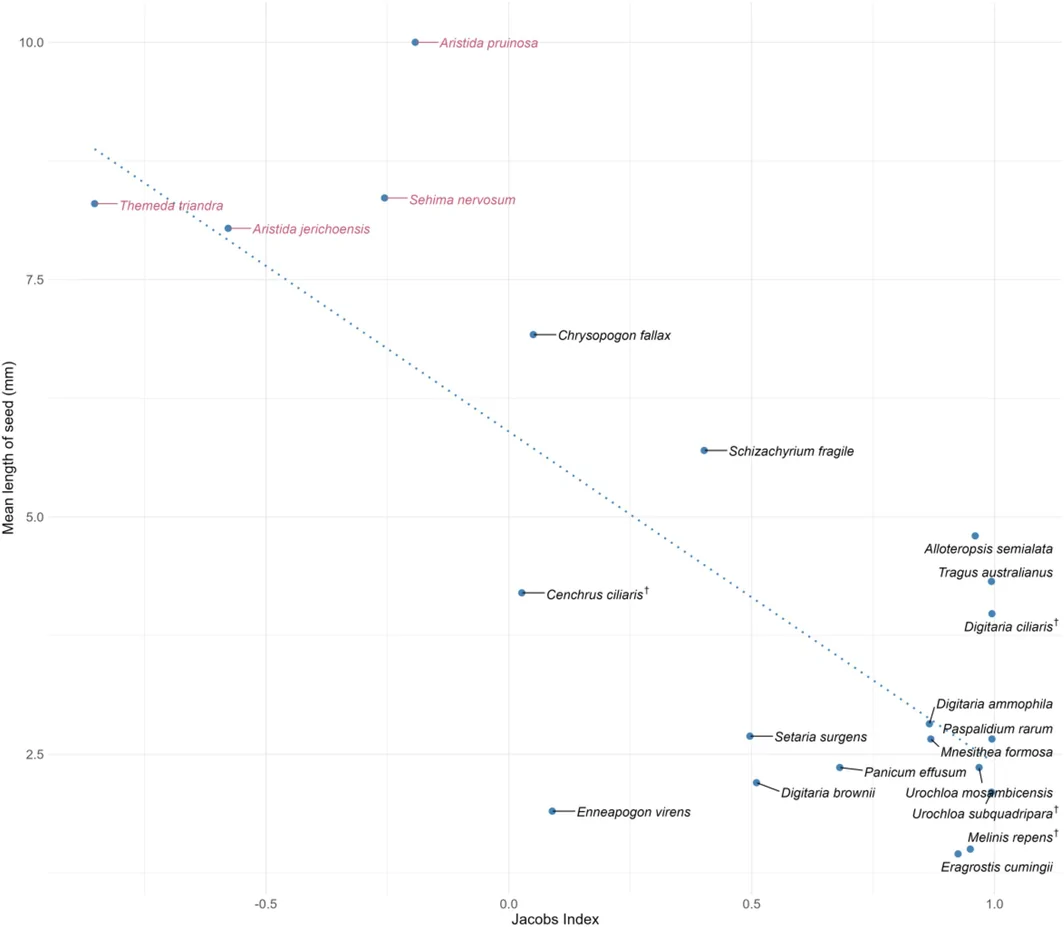
Relationship between Jacobs' Index and seed length of the seeds of 21 grass species that occur in the study area. Non‐native species depicted with †. Species with a negative Jacobs' Index are in red font.

## 4 Discussion

Our study found that black‐throated finches in the Desert Uplands demonstrate a selection preference for grass species with shorter seeds and that provide the greatest energy benefits. Observations of birds foraging identified a broad range of plant taxa consumed by black‐throated finches, comprising 33 grass species, as well as evidence of nectarivory. However, only a relatively small number of these species contributed to most of their diet, with seven grass genera contributing almost 68% of the diet based on foraging observations.

Foraging observations identified *Digitaria* spp., *Paspalidium* spp. and *Panicum* spp. as the three most desirable sources of black‐throated finch diet in the Desert Uplands. In the study area, three species of *Digitaria* have been recorded: *D. ciliaris* , *D. brownii* and *D. ammophila*, while only *Paspalidium rarum* and *Panicum effusum* have been identified from their respective genera. The high Jacobs' Index for these grasses indicates that black‐throated finches selectively target these species for consumption while their seeds are available. While they are not rare grasses throughout the study area, *D. ciliaris* (mean cover = 5.0% ± 6.2%), *D. brownii* (5.1% ± 4.9%), *D. ammophila* (1.4% ± 1.1%), *Paspalidium rarum* (1.2% ± 0.9%) and *Panicum effusum* (4.8% ± 6.2%) generally occur in low densities and were never the dominant grass species throughout black‐throated finch habitat. This is consistent with foraging behaviour recorded during previous studies in Townsville that also identified a strong preference for a small number of grasses (Mitchell 1996).

Conversely, the dominant grasses throughout the study area, namely *Heteropogon contortus* , *Themeda triandra* and *Aristida* spp. have a low Jacobs' Index, supporting the view that black‐throated finches target specific grasses, rather than consume seeds from those that are most readily available. These species also typically have larger seeds that may limit foraging by black‐throated finches due to their bill size and longer handling time, contributing to the lower Jacobs' index values. While these species were identified in the diet of black‐throated finches through DNA analysis (Dreis et al. in prep), black‐throated finches were rarely observed foraging on these species during the wet season, when seeds are often taken directly from the plant. Therefore, it appears that seeds from these grasses were likely to have been consumed from the ground and possibly whilst foraging in patches where more preferred grasses occur. Their relative contribution to black‐throated finch diet, as well as the Jacobs' Index, was typically higher for these species during the dry season. This may be a consequence of the declining availability of grass seeds during this period which may force birds to consume more readily available species (Woinarski and Tidemann 1991; Marone et al. 2017), but also supports the view that the black‐throated finch is able to diversify its diet during times of resource scarcity (van Osta et al. 2024).

Seasonal variation in the diet of black‐throated finches was difficult to detect from foraging observations alone. During the dry season, birds primarily feed on grass seeds from the ground with minimal seeds remaining on the plant, making it impossible to detect which species they are consuming. This may account for the seasonal difference in black‐throated finch diet based on foraging observations alone where fewer grass species were seen to be consumed during the dry season (*n* = 11) than the wet season (*n* = 31). However, the seasonal variation in the overall species richness observed here may also be due to some degree of sampling bias where birds may have been foraging in areas where they were not detected. Furthermore, results from DNA analysis of crop and faecal samples identified that black‐throated finch diet broadened and birds consumed more common grasses during the dry season (Dreis et al. in prep) which is consistent with previous studies of the black‐throated finch in Townsville (Mitchell 1996). The increased number of species consumed is likely to be a true seasonal dietary shift rather than a sampling artefact, as the pattern was consistent across both crop and faecal samples, with faecal DNA providing a direct measure of ingested diet that is not subject to the visual observation bias of the present study (Ando et al. 2020). Granivorous birds, including those in northern Australia, often exhibit these seasonal changes in diet in response to seed availability and nutritional requirements of grass seeds (Woinarski and Tidemann 1991; Garnett and Crowley 1994; Dostine and Franklin 2002; Moorcroft et al. 2002; Marone et al. 2017).

We identified a clear positive correlation between the preferred forage species of black‐throated finches and the levels of key nutritional factors provided by their seeds. Specifically, metabolisable energy and crude protein were typically higher in grass species with a higher Jacobs' Index. Metabolisable energy and crude protein are important for birds as they improve survival, growth, reproduction, egg development in addition to meeting caloric demands (Pinowska 1979; McWilliams 2001; Honarmand et al. 2010; Maute 2011; Pryke et al. 2012). This is consistent with optimal foraging theory that suggests that animals aim to maximise energy gain relative to effort and consider the trade‐off between factors including travel time, food type, density, nutritional value, and predation risk (MacArthur and Pianka 1966; Alonso et al. 1995; Sauter et al. 2006; King and Marshall 2022). To optimise energy intake, granivores may choose the grass seeds with higher caloric and nutrient content and/or those that are easiest to process (Díaz 1990, 1996; Dostine and Franklin 2002; Marone et al. 2022). Grasses with higher amounts of protein and metabolisable energy are likely to be more profitable for black‐throated finches, especially when their physical traits allow for efficient handling and processing (MacArthur and Pianka 1966; Grubb and Greenwald 1982; Nagy Koves Hrabar and Perrin 2002; Soobramoney and Perrin 2007; De León et al. 2014). Therefore, by displaying a preference for these grass seeds, black‐throated finches are able to maximise their energy consumption to allow more time for other activities including breeding activities and travelling between water sources and foraging and breeding habitats.

As well as the nutritional properties of grass seeds, the morphological characteristics may also be important for granivorous birds (Díaz 1996; Molokwu et al. 2011; Ríos et al. 2012; Titulaer et al. 2018). This appears to be the case for black‐throated finches who demonstrated a preference for grasses with shorter seeds, while abundant grass species such as *Themeda triandra* , *Aristida pruinosa* and *Sehima nervosum* with longer seeds were far less desirable. Seed size is important for granivorous birds because it influences the handling time and therefore affects foraging efficiency and energy intake (Schluter 1982; Díaz 1990; Nagy Koves Hrabar and Perrin 2002; Soobramoney and Perrin 2007; De León et al. 2014; Marone et al. 2022). The preference for smaller seeds, especially those with higher nutritional values, allows smaller birds such as the black‐throated finch to consume seeds quickly and increase their energy consumption.

## 5 Conclusion

This study identifies the dietary selection preferences of the black‐throated finch in the Desert Uplands bioregion. We found that black‐throated finches prefer a relatively small number of grass taxa that are characterised as having higher levels of metabolisable energy and crude protein as well as shorter seeds. By preferentially selecting these grasses, they can optimise their energy consumption by reducing handling time and maximising energy consumption. This allows birds to allocate more time to other activities such as breeding, drinking and avoiding predators. Seasonal differences in diet were difficult to detect from observations alone, due to the difficulty in identifying black‐throated finch diet during the dry season using this method. However, despite these limitations there were still trait‐based seed forage preferences observed between seasons. These findings provide valuable insights into the diet of this species and reinforce the importance of maintaining and managing habitats to promote a diversity of native grasses and especially those that are more profitable. The protection of areas of *Eucalyptus melanophloia* woodland habitat that contain higher cover of *Digitaria* spp., *Paspalidium rarum* and *Panicum effusum* is particularly important for the conservation of the black‐throated finch. Further research that explores the effect of grazing and fire on important diet sources as well as the response of black‐throated finches is required to understand how to improve the management of their habitat and maintain viable populations throughout their remaining distribution. Our study highlights the importance of understanding the selective foraging preferences for threatened species so that management actions can be implemented to increase the availability of these food sources.

## Author Contributions

**Brad Dreis:** conceptualization (lead), data curation (equal), formal analysis (equal), funding acquisition (lead), investigation (equal), methodology (equal), project administration (equal), resources (equal), validation (equal), visualization (equal), writing – original draft (lead), writing – review and editing (supporting). **James Guy Castley:** conceptualization (supporting), data curation (supporting), formal analysis (supporting), methodology (equal), supervision (lead), validation (supporting), visualization (supporting), writing – review and editing (lead). **John van Osta:** conceptualization (supporting), data curation (equal), formal analysis (supporting), funding acquisition (supporting), investigation (equal), methodology (supporting), project administration (equal), resources (equal), validation (equal), visualization (equal), writing – original draft (supporting). **Sarah Richardt:** formal analysis (supporting), software (supporting), supervision (supporting), validation (supporting), visualization (supporting), writing – review and editing (supporting). **Laetitia Smoll:** formal analysis (supporting), writing – original draft (supporting).

## Conflicts of Interest

The authors declare no conflicts of interest.

## Supporting information


**Data S1:** ece374425‐sup‐0001‐supinfo.xlsx.

## Data Availability

All the required data are uploaded as Supporting Information.
